# Metabolic Dysfunction and Oxidative Stress in Epilepsy

**DOI:** 10.3390/ijms18112365

**Published:** 2017-11-08

**Authors:** Jennifer N. Pearson-Smith, Manisha Patel

**Affiliations:** Department of Pharmaceutical Sciences, University of Colorado, Anschutz Medical Campus, Aurora, CO 80045, USA; Jennifer.pearson-smith@ucdenver.edu

**Keywords:** mitochondria, reactive oxygen species, seizures

## Abstract

The epilepsies are a heterogeneous group of disorders characterized by the propensity to experience spontaneous recurrent seizures. Epilepsies can be genetic or acquired, and the underlying mechanisms of seizure initiation, seizure propagation, and comorbid conditions are incompletely understood. Metabolic changes including the production of reactive species are known to result from prolonged seizures and may also contribute to epilepsy development. In this review, we focus on the evidence that metabolic and redox disruption is both cause and consequence of epileptic seizures. Additionally, we discuss the promise of targeting redox processes as a therapeutic option in epilepsy.

## 1. Introduction

Epilepsy is the fourth most common neurological condition, affecting approximately 65 million people worldwide [1]. It is a complex spectrum of disorders defined by the tendency to experience abnormal, highly synchronous brain activity known as a seizure. Known risk factors for the development of epilepsy fall into two main categories, those that have genetic origins and those that are acquired; however, in some cases the cause is unknown [1]. Currently available therapeutics include those that target ion channels or neurotransmitter systems [2]. These types of medications are effective at controlling seizure activity in about 60% of people with epilepsy but provide merely symptomatic relief, are ineffective in roughly 40% of people, and have the potential to exacerbate comorbidities [2,3]. There is therefore a great need to identify mechanisms that cause epilepsy and thus generate new therapeutic targets.

One promising avenue of research is the study of how metabolic dysfunction can contribute to seizures and exacerbate related sequalae such as neuronal loss and cognitive impairment. In this review, we will discuss the evidence suggesting the role of metabolic and redox alterations in genetic and acquired epilepsies.

## 2. Sources of Reactive Species and Oxidative Stress

**Figure 1:**
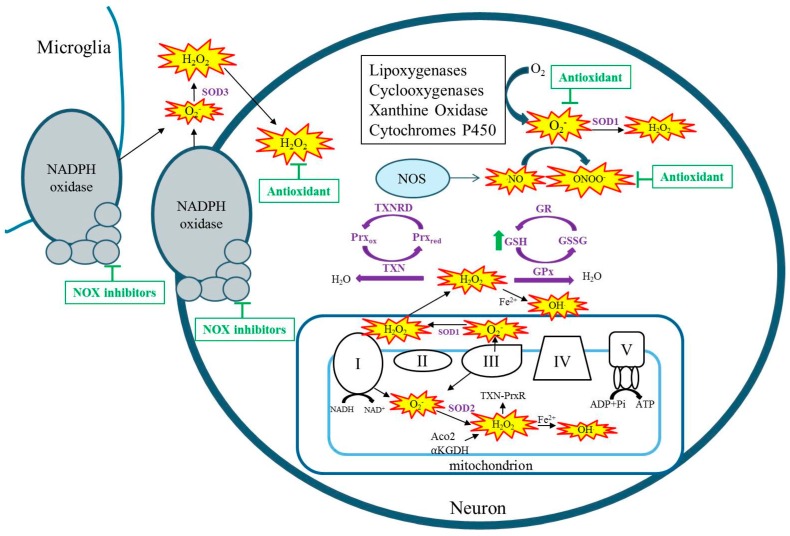
Cellular sources of reactive species (RS). RSs are denoted by yellow starbursts.Antioxidant systems and their detoxification of RS are denoted in purple and purple arrows, respectively. Therapeutic strategies for detoxification of seizure-induced RSS are denoted by green arrows, indicating elevating GSH or t-bars indicating inhibition by antioxidants or NOX inhibitors. NOS: nitric oxide synthase; GSH: reduced glutathione; GR: Glutathione reductase; GSSG: oxidized glutathione; GPx: Glutathione peroxidase; SOD: superoxide dismutase; TXN: thioredoxin; TXNRD: thioredoxin reductase; Prx_ox_: peroxiredoxin oxidized; Prx_red_: peroxiredoxin reduced; Aco2: aconitase; Α-KGDH: alpha-ketoglutarate dehydrogenase.

Free radicals are any chemical species with one or more unpaired electrons in the external orbit. Reactive species (RSs) i.e., reactive oxygen species (ROS) and reactive nitrogen species (RNS) collectively include singlet oxygen, superoxide (O_2_^−^), hydrogen peroxide (H_2_O_2_), nitric oxide (NO), hydroxyl radical (HO), and peroxynitrite (ONOO^−^). These RSs are produced by both enzymatic and non-enzymatic reactions and as a natural consequence of aerobic metabolism [4]. During aerobic metabolism, an infinitesimal percentage of oxygen consumed by mitochondria leaks from electron transport chain complexes I and III to form O_2_^−^, which can go on to form additional RSs such as H_2_O_2_ and OH (Figure 1) [5,6,7]. Whereas O_2_^−^ is not a strong oxidant itself, it can oxidize certain susceptible targets such as the labile iron–sulfur center of aconitase(s) [8,9]. In the kainate model of acquired epilepsy, the production of mitochondrial O_2_ has been demonstrated by the inactivation of mitochondrial, not cytosolic aconitase, and the production of H_2_O_2_ in isolated respiring mitochondria from the hippocampus [10,11]. Additionally, studies have demonstrated inhibition of α ketoglutarate dehydrogenase (α-KGDH) in a model of acquired epilepsy, inhibition of complex I in human temporal lobe epilepsy tissue as well as in immature and adult models of acquired epilepsy [12,13,14,15,16]. Regardless of the original source of RSs, when mitochondrial aconitase, α-KGDH, or complex I are inhibited, the result is additional RS production capable of creating vicious cycles of RS production in mitochondria [17,18,19,20]. Reactive species are also produced from enzymatic processes outside of the mitochondria such as by xanthine oxidase, nitric oxide synthase, cyclooxygenase, lipoxygenase, cytochrome p450, and nicotinamide adenine dinucleotide phosphate (NADPH) oxidases (Figure 1) [21,22]. Non-mitochondrial sources of ROS are activated by seizures, and targeting these enzymes has been shown to attenuate seizure-induced neurodegeneration [23,24,25,26]. When the production of RS exceeds the capacity of endogenous antioxidants to detoxify them, oxidative damage ensues.

The brain is particularly vulnerable to oxidant damage due to the abundance of mitochondria, high oxygen demand, poor repair capacity, and the presence of high concentrations of polyunsaturated fatty acids [27]. Oxidative stress can inflict damage to imperative cellular macromolecules including proteins, lipids, and DNA. Oxidation of proteins can change the structure, function, and activity of key enzymes [28]. Lipid peroxidation can compromise membrane structure leading to alterations in cell permeability and activity of membrane proteins, leading to hyperexcitability [29]. In the brain, oxidative damage to neural membranes can have profound effects on neurotransmitter uptake and release in addition to the maintenance of proper ionic gradients, again altering neuronal excitability [29]. Oxidative stress has been shown to contribute to the pathogenesis of a number of neurological conditions including Alzheimer’s disease, Parkinson’s disease, Huntington’s disease, stroke, and Amyotrophic Lateral Sclerosis [30,31]. In this review, we discuss the evidence which supports a key role of oxidative stress and mitochondrial function in the pathogenesis of the epilepsies. PubMed was searched for specific English terms including oxidative stress/damage in Alpers–Huttenlocher syndrome, oxidative stress, and Dravet syndrome. No restrictions regarding publication date, funder, or other article attributes were employed. The number of articles retrieved by such searches was relatively small (<100) and the relevance of the articles was assessed first by the abstract and second by the article text.

## 3. Oxidative Stress in Genetic Epilepsies

Epilepsy syndromes that are associated with genetic mutations are relatively rare, representing only a small fraction of all cases; however, this number is likely to increase as genetic testing becomes more commonplace. The genetic epilepsies are comprised of a heterogeneous group of disorders associated with diverse genetic mechanisms that can include de novo mutations to ion channel genes (channelopathies), chromosomal abnormalities, alterations to genes associated with brain malformations, and inherited or acquired mutations in mitochondrial DNA. In the following sections, we review genetic epilepsies that are associated with increased oxidative stress or metabolic dysfunction (summarized in Table 1).

### 3.1. Mitochondrial Encephalopathies

Mitochondrial DNA (mtDNA) mutations that result in epileptic phenotypes represent the strongest evidence for a link between epilepsy and oxidative stress or mitochondrial dysfunction. The most common of these include myoclonic epilepsy with ragged-red fibers (MERRF), Leigh syndrome, and Alpers–Huttenlocher syndrome. MERFF is caused by alterations to the *tRNA^Lys^* gene of the mtDNA, which affects complex I of the electron transport chain [32]. In cells cultured from patients with MERRF, there is evidence of increased ROS production, decreased ATP production, alterations to antioxidant gene expression, and oxidative damage [33]. Additionally, in cells harboring the genetic mutation associated with MERRF, mitochondrial calcium homeostasis was significantly altered, which could contribute to neuronal hyperexcitability [34]. Leigh syndrome is a neurodegenerative disease characterized by severe encephalopathy and refractory seizures. It is a genetically heterogeneous disorder that has been associated with over 35 mutations to mtDNA affecting any of the respiratory chain complexes. As such, those mutations affecting complex I result in increased ROS production and those mutations affecting complex V result in decreased ATP production [35,36,37]. In an animal model of Leigh syndrome, treatment with rapamycin attenuated neuropathological abnormalities and improved survival which the authors attributed to a metabolic shift away from glycolysis [38]. Another study suggested that genetic and pharmacological targeting of oxidative stress can attenuate neurodegeneration associated with Leigh syndrome [39]. Alpers–Huttenlocher syndrome (AHS) is caused by mutations to the mtDNA replicase, *POLG*, which disrupts mtDNA replication resulting in decreased mtDNA [40]. Neurologically, this disorder is characterized by refractory seizures, neuronal loss, and cognitive decline [41]. Decreased mtDNA can result in ATP depletion leading to energy failure and activation of apoptotic or necrotic cell pathways, which is evident in patients with AHS [42,43]. Additionally, ATP depletion is sufficient to induce neuronal hyperexcitability by altering sodium–potassium ATPase activity and decreasing neuronal membrane potential [44,45]. Thus, it would appear that oxidative stress contributes to seizures associated with mtDNA mutations or at the very least contributes to neuropathology.

### 3.2. Genetic Epilepsies Associated with Metabolic Dysfunction

Dravet syndrome (DS) is a rare and catastrophic epileptic encephalopathy that is characterized by intractable seizures and neurocognitive decline. In approximately 80% of patients, the disorder can be traced to de novo mutations in the *SCN1A* gene [46]. This gene encodes the sodium channel Nav1.1, and altered function of this channel seems to specifically affect GABAergic interneurons leading to decreased GABAergic tone and therefore increased hyperexcitability. Although direct evidence suggesting that oxidative stress contributes to seizure susceptibility or progression in DS is lacking, some evidence suggests that metabolism and mitochondrial defects may play a role. Perhaps the best evidence implicating metabolism as a therapeutic target in DS is the high degree of clinical efficacy of the ketogenic diet (KD). The KD provides alternative mitochondrial fuels and several studies have reported a greater than 50% reduction in seizure frequency with some patients attaining seizure freedom [47,48]. Additionally, stiripentol, in addition to its anticonvulsant action, inhibits a key enzyme for glycolysis and has shown promise as a novel medication to attenuate seizures in DS when given alone or in conjunction with other anticonvulsant drugs [49,50,51]. Although the mechanism(s) by which metabolically targeted therapies exert anticonvulsant effects are incompletely understood, one straightforward mechanism is by improving altered metabolic pathways. Indeed, in a small pilot study, fibroblasts obtained from patients with DS showed mitochondrial defects including severe reduction in mitochondrial complex III enzymatic activity and overall poor ATP output [52]. In a zebrafish model of DS, metabolic pathways were found to be perturbed leading to decreased glycolytic and oxygen consumption rates as well as downregulation of 5 genes associated with the glycolytic pathway [53]. Treatment with the KD returned altered metabolism to control levels in zebrafish and has been shown to attenuate seizure activity in this model [53,54]. Thus, a deeper understanding of how deficits in metabolism confer risk for seizures may prove useful in the development of novel therapeutics to treat genetic epilepsies.

Glucose transporter type 1 (GLUT1) deficiency syndrome is a rare metabolic disorder characterized by the appearance of recurrent seizures in early infancy. This disorder is caused by mutations to the *SLC2A1* gene, which encodes the protein required for glucose transport across the blood–brain barrier and into neurons. In addition to a wide array of seizure types, patients with this disorder typically present with cognitive delays and motor abnormalities. Exactly how GLUT1 deficiency results in seizures is unknown; however, as glucose is the main source of ATP in the brain, one obvious possibility is alterations to ATP production [55]. As mentioned previously, ATP depletion can alter neuronal membrane potential, leading to neuronal hyperexcitability [56]. Indeed, supplying the brain with alternative fuels, by use of the ketogenic or modified Atkins diet, is the most effective treatment to reduce seizures [57]. Although the effects of these diets on indices of oxidative stress have not been commonly reported in the literature, at least one study found that treatment with the modified Atkins diet significantly reduced oxidative damage to DNA and lipid peroxidation [58].

### 3.3. Genetic Epilepsies and Antioxidant Systems

Although research into the role of oxidative stress in human genetic epilepsies is rather limited, genetic animal models of epilepsy offer further evidence of oxidative stress in epileptic models. In genetically epilepsy-prone rats (GEPRs), endogenous antioxidant systems are perturbed which appears to contribute to an increased oxidative burden. Specifically, glutathione peroxidase (GPx) was found to be decreased and therefore less likely to protect against oxidative insults, resulting in decreased glutathione redox status, increased lipid peroxidation, and protein oxidization [59]. These oxidative markers were further exacerbated by kainate, and seizure activity was found to positively correlate with increased hippocampal oxidative stress markers [59].

Important information can also be gleaned by mouse models that are deficient in or overexpress endogenous antioxidants. For example, mice that are deficient in mitochondrial superoxide dismutase (SOD2) suffer from extensive mitochondrial dysfunction that culminates in increased mitochondrial oxidative stress, decreased ATP production, ataxia, and epilepsy before early postnatal death [60,61]. This suggests that oxidative stress and mitochondrial dysfunction can produce epilepsy and not be merely a consequence of seizures. Confirming the role of mitochondrial oxidative stress in the etiology of seizures, treatment with a catalytic antioxidant attenuated oxidative stress and seizure activity [61]. Even partial deficiency of SOD2 (*SOD2^−/+^*) was sufficient to increase the incidence of spontaneous and handling-induced seizure as a function of age, which correlated with increased mitochondrial oxidative stress [62]. Oxidation of targets that increase excitability is the most direct link between oxidative stress and epilepsy, and two such targets are the glial glutamate transporters, GLT-1 and GLAST. The uptake of extracellular glutamate by these transporters is driven by various electrochemical gradients, however specific “redox-sensing” elements have been identified, making these integral transporters sensitive to oxidation [63]. GLT-1 and GLAST were shown to be downregulated as a factor of age in *SOD2^−/+^* mice, which could account for their increased seizure vulnerability [62,63]. Overexpression of SOD2 was associated with decreased mitochondrial oxidative stress and protection from KA-induced hippocampal neurodegeneration [10]. These studies suggest an important role of excessive mitochondrial superoxide production as a contributing factor to the development of seizures and seizure-related neuropathology. 

While no human epilepsy syndrome resulting from SOD2 alterations has been identified, genetic mutations in another important antioxidant pathway, the thioredoxin system, have been associated with generalized epilepsy. Along with the glutathione system, the thioredoxin system serves as the cells major detoxifier of H_2_O_2_ (Figure 1) [64]. In fact, brain mitochondria have been shown to predominantly possess and utilize the thioredoxin–peroxiredoxin system for H_2_O_2_ detoxification [65]. Kudin and colleagues report a mutation in *thioredoxin reductase 1 (TXNRD1)* and its association with genetic generalized epilepsy [66]. In addition to reduced TXNRD1 activity, patient fibroblasts were less resistant to an H_2_O_2_ challenge [66]. Another study identified a patient suffering from an infantile-onset neurodegenerative disorder of which seizures were a prominent symptom [67]. Exome sequencing in this patient, uncovered a homozygous stop mutation in the mitochondrial form of thioredoxin (*TXN2*) [67]. Fibroblasts exhibited increased RS levels, inhibited oxidative phosphorylation and depletion of ATP, while restoration of *TXN2* or treatment with antioxidants protected cells against oxidative stress and damage [67]. These human studies suggest that epilepsy can arise due to reduced activity of the thioredoxin system. Interestingly, overexpression of thioredoxin in mice attenuates kainate-induced seizure severity and neuronal damage [68]. Thus, the proper functioning of this system may have important implications for epilepsy. Supporting this idea are numerous studies that demonstrate an association between selenium, an essential component of GPx and TXNRD, and seizures. Specifically, low selenium levels are associated with seizures in humans, whereas selenium supplementation in animal models attenuates seizure activity [69,70,71,72,73,74]. These antioxidant systems may therefore represent therapeutic targets.

## 4. Oxidative Stress in Acquired Epilepsies

The acquired epilepsy syndromes are those whose cause can be reasonably inferred from known or unknown neurological insults such as trauma, infection, ischemic stroke, or status epilepticus (SE). The precipitating brain injury leads to a process known as epileptogenesis in which cellular and biochemical changes occur and spontaneous recurrent seizures (SRSs) arise after a latent or seizure-free period of weeks to years. These initiating brain injuries particularly stroke, trauma and SE are known to increase oxidative stress [75,76,77]. Oxidative stress can contribute to neuronal hyperexcitability and neuronal cell death through numerous mechanisms including oxidative damage to membrane proteins such as neurotransmitter receptors and ion channels [28]. The acquired epilepsies and animal models thereof have consistently shown that oxidative stress can result from seizure activity and exacerbate consequences of seizures such as neuronal loss and cognitive impairment. The role of oxidative stress in acquired epilepsies is reviewed briefly here; however, for a more detailed review please see [78]. In patients with temporal lobe epilepsy (TLE), antioxidant systems such as glutathione and SOD are altered, indicative of ongoing oxidative stress [79,80,81]. Indeed, oxidative damage to biomolecules has been detected in surgically resected epileptic brain tissue and suggested to contribute to neuronal hyperexcitability and degeneration [13,80,82,83]. Animal models of acquired epilepsy have shown seizure-induced oxidative damage to vulnerable mitochondrial and hippocampal proteins (complex I and aconitase, etc.), mitochondrial DNA (8-OhdG), and various lipids in the hippocampus, the site of characteristic and selective seizure-induced neurodegeneration at times preceding overt neuronal death [10,11,14,84,85,86,87]. In epilepsy, key mediators of neuronal death are necrosis initiated by glutamate excitotoxicity and apoptosis [88,89,90,91,92,93]. Oxidative stress and mitochondria are contributors to glutamate excitotoxicity as well as apoptotic cell death [92,93,94,95]. The intrinsic (mitochondrial) pathway of apoptosis is initiated by intracellular abnormalities, such as DNA damage, calcium overload, and oxidative stress [96,97]. If sufficient to induce loss of the mitochondrial membrane potential, this signals the subsequent release of pro-apoptotic proteins (cytochrome c, apoptosis-inducing factor, etc.) and downstream activation of caspase-dependent and caspase-independent cell death [98]. The role of oxidative stress in seizure-induced neuronal cell death is well established [99,100,101,102,103,104]. In fact, treatment with various compounds that act to decrease oxidative stress (antioxidants, NADPH oxidase inhibitors, etc.) have been demonstrated to protect against seizure-induced neuronal death [24,99,100,101,105,106]. Neuronal death, particularly in the hippocampus, is a common feature of acquired epilepsy and is thought to contribute to cognitive dysfunction [107]. Importantly, attenuation of oxidative damage in models of acquired epilepsy appears to protect against cognitive dysfunction as well as confer neuroprotection [85,105,106]. Thus, oxidative damage results from seizure activity in acquired epilepsy and appears to contribute to seizure-induced neuronal loss and cognitive dysfunction.

## 5. Oxidative Stress as a Therapeutic Target

Targeting of oxidative stress to attenuate seizures and related sequalae has largely been limited to studies of animal models. Vitamin E has been shown to have antioxidant properties and has shown promise in attenuating oxidative stress and seizure parameters in various animal models but not others [108,109]. This animal data is supported by clinical data suggesting that Vitamin E can significantly reduce oxidative stress markers and seizure frequency [110,111]. Treatment with Vitamin E is somewhat controversial, however, as other studies have found no significant benefits on seizure frequency, and the dosage needed to achieve therapeutic concentrations is relatively high. Indeed, clinical data may be sparse, as it is relatively difficult for large antioxidant molecules to pass the blood–brain barrier, and antioxidants that act stoichiometrically to quench free radicals need to be given frequently and in large doses. One way around this is to treat with compounds that act to bolster the endogenous antioxidant systems (Figure 1). For example, a recent study demonstrated that co-treatment with *N*-acetylcysteine and sulforaphane, which act to increase glutathione levels, protected against brain oxidative stress, reduced seizure frequency, and attenuated neuronal loss and cognitive deficits [105]. Importantly, these compounds are already approved for human use in other conditions. Another potential therapeutic option is to use catalytic antioxidants that act enzymatically to scavenge free radicals to decrease oxidative stress (Figure 1). Although these types of compounds have not been shown to attenuate seizure activity, likely due to short treatment periods in animal models, they have shown excellent neuroprotective profiles and attenuation of seizure-induced cognitive dysfunction and may have potential as adjunctive therapies to current anti-seizure medications [61,106]. Similarly, inhibition of NADPH oxidase has been demonstrated to attenuate seizure-induced cell death [23,24]. Given that ROS may play a physiological role in cell signaling, any therapies targeting these pathways need to be thoroughly evaluated for deleterious side effects [112].

## 6. Conclusions

In this review, we have presented an overview of the evidence suggesting that oxidative stress plays a key role in genetic and acquired epilepsies. Targeting oxidative stress may provide a novel therapeutic to attenuate seizure activity and associated sequalae such as neuronal loss and cognitive impairment. Future research in the area will further elucidate the contribution of specific reactive species and mechanisms that transform the normal brain to one prone to hyperexcitability, thus providing novel therapeutics in conjunction with or in replacement of current anti-seizure medications.

## Figures and Tables

**Table 1:** Select studies implicating oxidative stress and metabolic dysfunction in select epilepsies associated with a genetic cause.

Disorder	Gene Mutation	Resulting Dysfuntion	Finding	Citation
MERFF	*tRNA^Lys^*	Complex I	Decreased ATP, increased ROS, altered antioxidant gene expression, alterations to calcium homeostatsis	[32,33,34]
Leigh syndrome	Various mtDNA mutations	Complex I, V	Increased ROS, decreased ATP	[35,36,37]
AHS	*POLG*	Decreased mtDNA, Complex IV	Increased apoptosis and necrosis potentially modulated by mito pathways	[40,42]
Dravet Syndrome	*SCN1A*	Nav1.1	In zebrafish—decreased glycolytic and oxygen consumption rates, downregulation of glycolytic pathway	[53]
Glut1 deficiency	*SLC2A1*	Glucose transport into brain	Increased oxidative DNA damage, increased lipid peroxidation—attenuated by modified Atkins diet	[58]

MERFF: Myoclonic epilepsy with ragged-red fibers; AHS: Alpers-Huttenlocher syndrome; ROS: reactive oxygen species.
